# Lifetime survival and medical costs of lung cancer: a semi-parametric estimation from South Korea

**DOI:** 10.1186/s12885-020-07353-8

**Published:** 2020-09-03

**Authors:** Hae-Young Park, Jinseub Hwang, Do-Hyang Kim, Soo Min Jeon, Sun Ha Choi, Jin-Won Kwon

**Affiliations:** 1 College of Pharmacy and Research Institute of Pharmaceutical Sciences, Kyungpook National University, Daegu, 41566 South Korea; 2 Division of Mathematics and Big Data Science, Daegu University, Gyeongsan-si, 38453 South Korea; 3 Lung Cancer Center, Kyungpook National University Chilgok Hospital, Daegu, 41404 South Korea

**Keywords:** Lung cancer, Disease burden, Mortality, Costs, Survival analysis

## Abstract

**Background:**

It is essential to have information on the disease burden of lung cancer at an individual level throughout the life; however, few such results have been reported. Thus, this study aimed to assess the lifetime disease burden in patients with lung cancer by assessing various factors, such as survival, years of life lost (YLL) and medical expenditure in South Korea based on real-world data and extrapolation.

**Methods:**

Newly diagnosed lung cancer patients (*n* = 2919) in 2004–2010 were selected and observed until the end of 2015 using nationwide reimbursement claim database. The patients were categorised into the Surgery group, Chemo and/or Radiotherapy group (CTx/RTx), and Surgery+CTx/RTx according to their treatment modality. Age- and sex-matched control subjects were selected from among general population using the life table. The survival and cost data after diagnosis were analysed by a semi-parametric method, the Kaplan–Meier analysis for the first 100 months and rolling extrapolation algorithm for 101–300 months. YLL were derived from the difference in survival between patients and controls.

**Results:**

Lifetime estimates (standard error) were 4.5 (0.2) years for patients and 14.5 (0.1) years for controls and the derived YLL duration was 10.0 (0.2) years. Lifetime survival years showed the following trend: Surgery (14.2 years) > Surgery+CTx/RTx (8.5 years) > CTx/RTx group (3.0 years), and YLL were increased as lifetime survival years decreased (2.3, 8.7, 12.2 years, respectively). The mean lifetime medical cost was estimated at 30,857 USD/patient. Patients in the Surgery group paid higher treatment cost in first year after diagnosis, but the overall mean cost per year was lower at 4359 USD compared with 7075USD of Surgery+CTx/RTx or 7626USD of CTx/RTx group.

**Conclusions:**

Lung cancer has resulted in about 10 years of life lost in overall patients. The losses were associated with treatment modality, and the results indicated that diagnosing lung cancer in patients with low stage disease eligible for surgery is beneficial for reducing disease burden in terms of survival and treatment cost per year throughout the life.

## Background

The survival rate of lung cancer patients has improved over the years with newer treatments; however, the average five-year survival rate of lung cancer remains < 20% considering all stages, which is substantially low compared to other major cancers and is the leading cause of cancer deaths. In addition, the global prevalence of lung cancer has increased by 29% between 2005 and 2015 due to growth and aging in the overall population [1, 2]. The increasing rate of incidence is dependent on the region, smoking habits, gender and socio-environmental status, and considering the trend of lung cancer prevalence, this rate was predicted to continue to increase in next10 years [3]. With this high mortality rate and increasing incidence, lung cancer is a worldwide public health problem, and information on the burden of lung cancer will be critical to the development of new drugs and framing of health policies in the future.

Recently, based on the South Korean longitudinal claim data, we reported relative mortality and medical expenditure data for lung cancer patients in the first 5 years after diagnosis [4]. However, the influence of the remaining lifetime and future medical costs on the burden of lung cancer is uncertain. Furthermore, if lifetime survival and cost data can be available beyond the initial 5 years of treatment, the average cost of treatment per year of life can be calculated. Such information would be instrumental in assessing treatment efficiency and prioritising budget allocation for target patient group.

However, not many studies have analysed the disease burden over a patient’s lifetime because an extrapolation process is required to implement this analysis, and extrapolation inevitably has uncertainty. For lung cancer, we could find only one study by Yang et al. that analysed the national cohort data for 66,535 Taiwanese patients in 1998–2010 and presented lifetime survival and cost of lung cancer by pathological subtype [5]. They extrapolated survival probabilities using the method suggested by Hwang et al. in 1999 [6]; this method was aimed at predicting the survival of the target cohort using the survival information of the matched reference group by assuming an excess constant hazard between the matched reference group and the target cohort. However, the method has a limitation in that it has an assumption that the excess hazard of the patients group remains constant for the entire extrapolation period. Hwang et al. reported a new algorithm of rolling extrapolation using restricted cubic splines models in 2017 that complemented the limitations of the extrapolation method proposed in 1999 [7] and proved that the new estimation method is superior to the existing method through simulation studies. The new rolling extrapolation can be applied to any country irrespective of the health care system, provided that actual data is available for a given period of time. However, so far, to our knowledge, no research using the new method to assess the disease burden for patients with lung cancer has been published.

Thus, we conducted this study to estimate following items; (1) lifetime survival and medical expenditure in patients with lung cancer based on the real data and extrapolation, (2) years of life lost (YLL) of patients with lung cancer according to treatment modality, from which the stage of the disease could be indirectly estimated, (3) yearly medical cost (YMC) and sensitivity analysis to evaluate the uncertainty of the extrapolation.

## Methods

### Database and study population

This study used the same database to define study population as used in our preceding research, the National Health Insurance Service-National Health Screening Cohort data, 2002–2015 [4]. We first selected 7502 lung cancer patients who had diagnosis code of lung cancer (e.g., C33: malignant neoplasm of the trachea; C34: malignant neoplasm of the bronchus and lung) during the 2004–2010 period. Finally, 2919 patients were selected after excluding 1371 patients who had preceding diagnosis of other cancers in 2002–2003 and 3212 patients who survived for more than a year without any record of treatment for lung cancer. The source database did not provide information on disease stages. Thus, Patient subgroups were defined as follows according to the treatment modality in the first year after diagnosis instead of disease stage: Surgery-only group (*n* = 426), chemo and/or radiotherapy group (CTx/RTx, *n* = 1405) without surgery, best supportive care (BSC, *n* = 792) and Surgery and chemo and/or radiotherapy group (Surgery + CTx/RTx, *n* = 296), which included patients who underwent pre-operative adjuvant chemo and/or radiotherapy (*n* = 213) and post-operative adjuvant chemo and/or radiotherapy (*n* = 83) (Supplement Figure A.1). The CTx/RTx group include patients who received chemotherapy or target therapy or immunotherapy, combined with or without radiotherapy, and the BSC group is defined as those who received no anti-tumor therapy.

### Analysis of survival during the follow-up period

During the follow-up period (100 months after diagnosis) of the patient group, survival probability was estimated using the Kaplan–Meier method. To estimate the survival of the reference group, age- and sex-matched target subjects among the general population were selected based on the life table of the Republic of Korea from 1996 to 2017 [8]. Similarly to the patient group, survival times for the selected controls were generated using the Monte Carlo method, and survival probability for the control group was estimated using the Kaplan–Meier method [7]. The BSC group among subgroups in the patients could not match the control group due to its short follow-up period and was thus excluded from subgroup analysis for lifetime estimation.

### Extrapolation of survival until the end of lifetime

Time of ending extrapolation for lifetime analysis was set at 300 months after diagnosis. Survival of the patient group from 101 months until the end of extrapolation was estimated based on the rolling extrapolation algorithm method proposed by Hwang et al. (2017) [7]. Hwang et al. presented their basic extrapolation method in 1999 [6]; this method was aimed at estimating survival probabilities after the follow-up period using an estimated regression coefficient. The coefficient was obtained from a linear regression model based on the follow-up time and the logit transformed relative survival between a patient group and a control group. Rolling extrapolation algorithm was improved based on the basic method presented by Hwang et al. in 1999, and could reflect more recent survival trend during the extrapolation. The iSQoL (version 4.2, Tsuey-Hwa Hu) [9], an open package of R programs, was used to compute the extrapolation of survivals and medical expenditure.

### YLL estimates

YLL was estimated based on survival estimates and their standard errors (SEs) for the control and patient groups, which were independent of each other during the 300 months. The estimation was performed according to the following formula: $$ \hat{\boldsymbol{YLL}}={\hat{\boldsymbol{S}}}_{\boldsymbol{reference}}-{\hat{\boldsymbol{S}}}_{\boldsymbol{patient}}\ \left(\hat{\boldsymbol{S}}: Mean\ survival\ estimates\right), $$$$ {\hat{\boldsymbol{SE}}}_{\boldsymbol{YLL}}=\sqrt{{\hat{\boldsymbol{SE}}}_{\boldsymbol{reference}}^{\mathbf{2}}+{\hat{\boldsymbol{SE}}}_{\boldsymbol{patient}}^{\mathbf{2}}} $$

### Medical expenditure analysis

First, medical expenditure during the follow-up period (100 months) was calculated based on the Kaplan–Meier sample average estimator method [10] with the following formula: $\mathrm{Cost}={\sum}_{\mathrm{t}=1}^{100}\mathrm{S}\left(\mathrm{t}\right){\mathrm{C}}_{\mathrm{t}}$ [S(t): survival probability in month t; C(t): the mean cost in the month t among the survived patients in month t]. Medical expenditure after the follow-up period until the time of ending extrapolation was estimated using a rolling extrapolation survival-adjusted cost (RESAC) estimator proposed by Hwang et al. (2017) [7]. The average yearly medical cost (YMC) during lifetime, which was defined as the ratio lifetime medical expenditure (C) to lifetime survival (S), wherein C and S are not independent of each other, was estimated by the following Taylor expansion approximation method [11]:

$$ {\displaystyle \begin{array}{c}\hat{YMC}\approx \frac{\hat{C}}{\hat{S}}-\frac{Cov\left(\hat{C},\hat{S}\right)}{{\hat{S}}^2}+\frac{Var\Big(\hat{S}\Big)\hat{C}}{{\hat{S}}^3}\ \left( Cov: covariance, Var: Variance\right),\\ {}\mathrm{SE}\Big(\hat{YMC\Big)}=\sqrt{\frac{{\hat{C}}^2}{{\hat{S}}^2}\left[\frac{Var\left(\hat{C}\right)}{{\hat{C}}^2}-2\frac{Cov\left(\hat{C},\hat{S}\right)}{\hat{C}\hat{S}}+\frac{Var\left(\hat{S}\right)}{{\hat{S}}^2}\right]}\end{array}} $$

The covariance $Cov\left(\hat{C},\hat{S}\right)$ was assumed to be the same value as the covariance derived from the data of medical expenditure and survival estimates during the follow-up period. All expenses during the follow-up period were adjusted based on the Consumer Price Index for medical care in 2017 and presented in US dollars based on the exchange rate assumption of 1100 Korean Won/USD. Discounting was not considered for the extrapolated cost because discount was not applied to the extrapolated survival.

### Statistics

Survival and medical expenditures were presented as mean estimates of the survival period and medical expenditure with SE values in the follow-up period and during the lifetime, respectively. SE was estimated using the bootstrapping method with 100 random samples. For validating the extrapolation method, the survival estimates for 10 years based on a semi-parametric method (5-year follow-up + 5-year extrapolation) were compared with Kaplan–Meier survival estimates with a follow-up period of 10 years. Sensitivity analysis was conducted to evaluate how the length of follow-up period (varied to 60, 80, 120 months, or the last observation month) and ending time of extrapolation (varied to 240 or 360 month) affected the analysis results. All analyses were conducted using the R Statistical Software (version 3.5.2; R Foundation for Statistical Computing, Vienna, Austria).

## Results

### Characteristics of the study population

The average age (SE) of total patients was 67.1 years (9.2 years). In BSC, CTx/RTx, Surgery and Surgery+CTx/RTx subgroups, average ages at diagnosis were 73.2 years (7.7 years), 65.8 years (8.6 years), 63.7 years (8.9 years) and 62.1 years (8.3 years), respectively. The incidence of lung cancer tended to increase slightly during 2004–2010. The proportion of smokers was about 51%, and the average CCI score was 2.3 ± 1.3 (Table 1).

**Table 1:** Characteristics of the study population^a^

Number of patients (%)	Surgery	Surgery+CTx/RTx	CTx/RTx	BSC	Total
Total	426 (14.6)	296 (10.1)	1405 (48.1)	792 (27.1)	2919 (100.0)
Males	313 (73.5)	221 (74.7)	1135 (80.8)	611 (77.2)	2280 (78.1)
Age (years)					
Mean (SE)	63.7 (8.9)	62.1 (8.3)	65.8 (8.6)	73.2 (7.7)	67.1 (9.2)
Median (IQR)	64.5 (13.0)	62.0 (12.0)	67.0 (12.0)	74.0 (10.0)	68.0 (13.0)
Year at diagnosis					
2004	58 (13.6)	30 (10.1)	171 (12.2)	132 (16.7)	391 (13.4)
2005	46 (10.8)	34 (11.5)	195 (13.9)	105 (13.3)	380 (13.0)
2006	44 (10.3)	38 (12.8)	197 (14.0)	105 (13.3)	384 (13.2)
2007	59 (13.9)	54 (18.2)	210 (15.0)	109 (13.8)	432 (14.8)
2008	75 (17.6)	50 (16.9)	195 (13.9)	124 (15.7)	444 (15.2)
2009	72 (16.9)	43 (14.5)	204 (14.5)	98 (12.4)	417 (14.3)
2010	72 (16.9)	47 (15.9)	233 (16.6)	119 (15.0)	471 (16.1)
Smoking status					
Missing	5 (1.2)	2 (0.7)	22 (1.6)	17 (2.2)	46 (1.6)
Non-smoker	216 (50.7)	148 (50.0)	613 (43.6)	404 (51.0)	1381 (47.3)
Smoker^**b**^	205 (48.1)	146 (49.3)	770 (54.8)	371 (46.8)	1492 (51.1)
Charlson comorbidity patient score					
Mean (SE)	2.3 (1.3)	2.1 (1.2)	2.3 (1.3)	2.6 (1.4)	2.3 (1.3)
Median (IQR)	2.0 (2.0)	2.0 (2.0)	2.0 (2.0)	2.0 (3.0)	2.0 (2.0)

*BSC* best supportive care, *CTx/RTx* chemotherapy and/or radiation therapy, *IQR* interquartile range, *SE* standard error

^a^The most recent health screening results based on the time of diagnosis

^b^Current smoker or ex-smoker

### Lifetime survival and YLL estimation

**Fig. 1:**
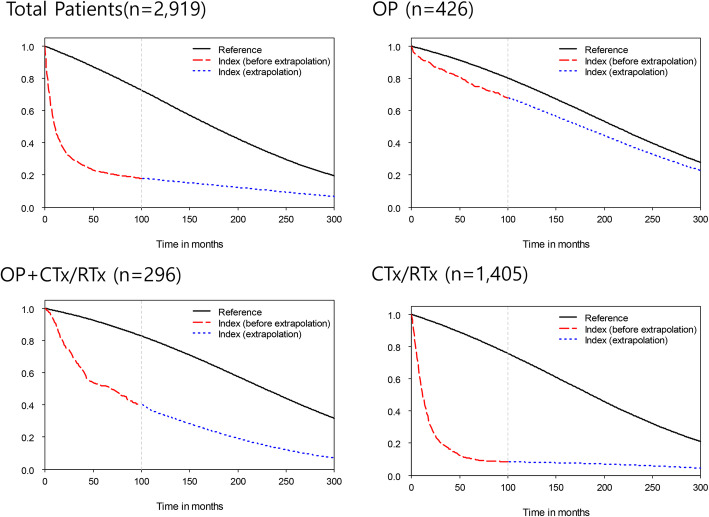
Survival probability of lung cancer patients over 25 years. CTx/RTx; chemotherapy and/or radiation therapy, OP; surgery

Lifetime survival probabilities of both patient and control groups were estimated as shown in Fig. 1. Except for a slight fall in the initial time, the survival curve of patients in the Surgery group appeared close and almost parallel to that of the control group. The curve of the CTx/RTx group dropped sharply in the first few years and gradually decreased, indicating the greatest difference from the curve of the control group. The average survival period of total patients was estimated to be 2.48 years during the follow-up period, and the estimated life expectancy was 4.53 years over a 25-year time horizon. Among the subgroups, Surgery and Surgery+CTx + RTx groups had relatively longer survival periods of 14.19 years and 8.51 years, respectively; however, CTx/RTx and BSC groups showed lower values as 3.01 years and 0.37 year, respectively. The estimated life expectancy of the control group was estimated as 14.51 years, and the average YLL was assumed to be 9.99 years in overall patients. YLL increased with decreasing survival estimates and were 2.30 years, 8.74 years, 12.16 years for Surgery, Surgery+CTx/RT, CTx/RTx groups, respectively (Table 2).

**Table 2:** Life expectancy and YLL analysis results for patients with lung cancer

Year (Standard error)	Patients with lung cancer		Control group	Years of life lost (YLL)
	ST^a^	eST ^b^	eST ^b^	
Total (*n* = 2919)	2.48 (0.06)	4.53 (0.21)	14.51 (0.10)	9.99 (0.23)
Surgery (*n* = 426)	6.73 (0.13)	14.19 (0.91)	16.49 (0.22)	2.30 (0.94)
Surgery+CTx/RTx (*n* = 296)	5.09 (0.20)	8.51 (1.00)	17.25 (0.22)	8.74 (1.02)
CTx/RTx (*n* = 1405)	1.87 (0.06)	3.01 (0.20)	15.18 (0.13)	12.16 (0.24)
BSC (*n* = 792)	0.37 (0.01)	NA	NA	NA

*BSC* best supportive care, *CTx/RTx* chemotherapy and/or radiation therapy, *eST* estimated survival time, *ST* survival time

^a^Actual survival time based on the real data from a 100-month follow-up period

^b^Estimated survival time based on the actual survival time during the real follow-up period (100 months) and extrapolated life expectancy for the next 200 months

### Lifetime medical expenditure

**Fig. 2:**
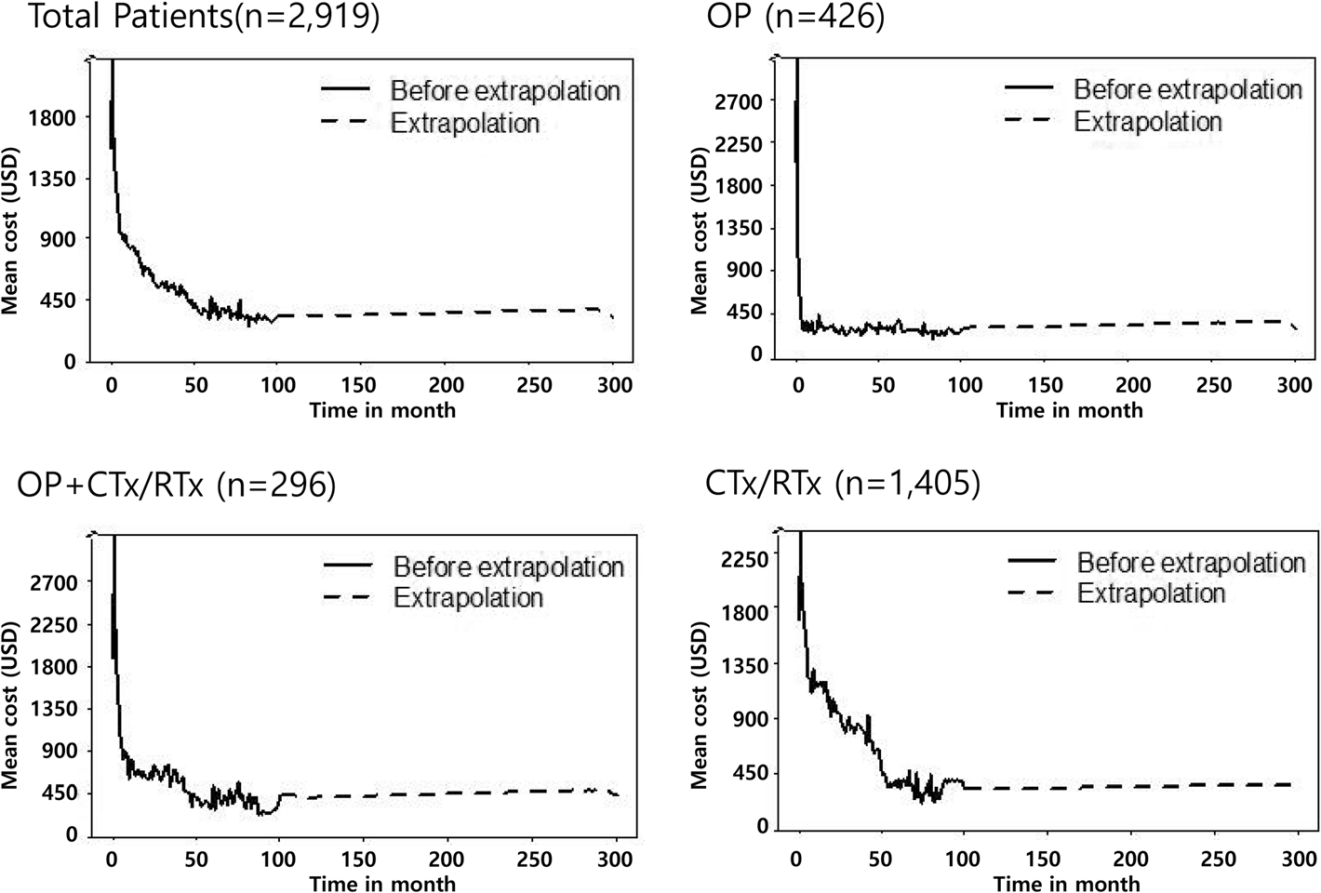
Actual and extrapolated medical expenditure in lung cancer patients over 25 years. CTx/RTx; chemotherapy and/or radiation therapy, OP; surgery

Medical expenditure incurred overall or for specific subgroups as shown in Fig. 2. Patients in the Surgery group paid high initial cost, which subsequently declined sharply and then remained low. The Surgery+CTx/RTx or CTx/RTx group had longer initial periods of high treatment costs and expenditure during the extrapolation period was higher compared with the Surgery group. The actual medical expenditure during follow-up period (100 months) was 42,384 USD, 30,843 USD and 25,255 USD for Surgery+CTx/RTx, Surgery, and CTx/RTx groups, respectively. The lifetime medical expenditure was estimated to be 1.2–2.0 times of true actual expenditure during the follow-up period. YMC showed the following trend: CTx/RTx (7626 USD/year) > Surgery+CTx/RTx > (7075 USD/year) > Surgery group (4359 USD/year) (Table 3).

**Table 3:** Lifetime medical expenditure for patients with lung cancer

USD^a^ (Standard error)	Patients with lung cancer		Cost ratio B to A	Yearly medical cost (YMC) in USD
	ME^b^ (A)	eME^c^ (B)		
Total (*n* = 2919)	22,130 (506)	30,857 (1156)	1.39	5393 (399)
Surgery (*n* = 426)	30,843 (1147)	61,592 (4722)	2.00	4359 (434)
Surgery+CTx/RTx (*n* = 296)	42,384 (1699)	60,640 (4894)	1.43	7075 (1012)
CTx/RTx (*n* = 1405)	25,255 (578)	30,079 (1261)	1.19	7626 (756)
BSC (*n* = 792)	5562 (187)	NA	–	12,042 (560)

*BSC* best supportive care, *CTx/RTx* chemotherapy and/or radiation therapy, *eME* estimated medical expenditure, *ME* medical expenditure

^a^Applied exchange rate: 1100 Korea Won/USD

^b^Actual medical expenditure based on real data analysis during a 100-month follow-up period

^c^Estimated medical expenditure based on the actual expenditure during the real follow-up period (100 months) and extrapolated costs for the next 200 months

### Validation results

The 10-year survival estimated by the 5-year follow-up and 5-year rolling extrapolation algorithm was very similar to the results estimated by a 10-year follow-up with the Kaplan–Meier method. The mean survival (SE) of total patients was 2.69 years (0.08 years) for semi-parametric method and 2.76 years (0.07 years) for 10-year follow-up with the Kaplan–Meier method, and the subgroups also showed similar results for the two methods (Supplement Table A.1).

### Sensitivity analysis according to follow-up period

The follow-up period affected the results of lifetime survival and YMC. As the follow-up period was reduced to 60 months, YMC increased to 127% of base analysis value, and when the period was extended to 143 months in total patients, it decreased to 89% of base analysis value. The most sensitive results were shown in the CTx/RTx group with 10,150 USD/year of YMC, which was increased to 133% of base analysis value in the follow-up period of 60 months (Table 4). Change of lifetime horizon (240 months or 360 months) did not affect YMC significantly, as the variance was within ±5% of the base results from a lifetime horizon of 300 months (Supplement Table A.2, Table A.3).

**Table 4:** Sensitivity analysis according to follow-up period

Survival: year (SE), Cost: USD (SE)	Follow-up month	Survival		Medical expenditure		Yearly medical cost (YMC) in USD	Deviation % in YMC
		ST during real follow-up	eST during lifetime (300 months)	ME during real follow-up	eME during lifetime (300 months)		
Total (*n* = 2919)	100 (base)	2.48 (0.06)	4.53 (0.21)	22,130 (506)	30,857 (1156)	5393 (399)	100%
	60	1.82 (0.04)	3.67 (0.22)	19,544 (399)	29,714 (1164)	6851 (576)	127%
	80	2.16 (0.05)	4.50 (0.23)	20,928 (417)	31,262 (1171)	5782 (440)	107%
	120	2.76 (0.07)	4.51 (0.20)	22,886 (539)	28,918 (1087)	4835 (357)	90%
	Last (143 month)	3.07 (0.08)	4.85 (0.23)	23,609 (544)	30,058 (1305)	4779 (388)	89%
Surgery (*n* = 426)	100 (base)	6.73 (0.13)	14.19 (0.91)	30,843 (1147)	61,592 (4722)	4359 (434)	100%
	60	4.32 (0.07)	11.19 (1.76)	22,551 (769)	53,539 (5995)	4917 (924)	113%
	80	5.56 (0.09)	13.92 (1.11)	27,270 (1021)	63,507 (5528)	4596 (538)	105%
	120	7.82 (0.17)	14.33 (0.85)	33,271 (1347)	55,003 (5003)	3852 (417)	88%
	Last (143 month)	9.03 (0.21)	15.00 (0.72)	36,892 (1932)	64,472 (7368)	4299 (533)	99%
Surgery+CTx/RTx (*n* = 296)	100 (base)	5.09 (0.20)	8.51 (1.00)	42,384 (1699)	60,640 (4894)	7075 (1012)	100%
	60	3.56 (0.10)	9.93 (1.22)	35,835 (1642)	71,326 (6374)	7260 (1093)	103%
	80	4.39 (0.13)	9.08 (0.96)	39,406 (1680)	65,710 (5290)	7235 (958)	102%
	120	5.73 (0.23)	9.53 (1.07)	45,035 (2668)	59,804 (6702)	6217 (994)	88%
	Last (137 month)	6.20 (0.29)	10.12 (1.01)	45,970 (2239)	57,512 (5237)	5613 (764)	79%
CTx/RTx (*n* = 1405)	100 (base)	1.87 (0.06)	3.01 (0.20)	22,255 (578)	30,079 (1261)	7626 (756)	100%
	60	1.56 (0.05)	2.01 (0.11)	24,244 (571)	27,740 (888)	10,150 (835)	133%
	80	1.73 (0.06)	2.78 (0.23)	24,684 (568)	28,937 (1233)	8058 (947)	106%
	120	2.00 (0.07)	2.78 (0.21)	25,443 (643)	28,214 (1076)	7176 (824)	94%
	Last (142 month)	2.13 (0.08)	2.94 (0.20)	25,513 (572)	26,631 (701)	6337 (617)	83%

*BSC* best supportive care, *CTx/RTx* chemotherapy and/or radiation therapy, *eME* estimated medical expenditure, *eST* estimated survival time, *ME* medical expenditure, *ST* survival time

^a^Applied exchange rate: 1100 Korea Won/USD

## Discussion

This study assessed lifetime disease burden of lung cancer at an individual level based on real-world data and extrapolation. Compared to general population, patients with lung cancer were found to have an average YLL of about 10 years over their entire lifetime. Since the treatment modality chosen differed depending on the disease stage or pathological subtype, we assumed that the disease burden would differ depending on the treatment method. Thus, the we performed subgroup analyses in this study according to treatment modality, and the study results revealed a significant difference in the pattern of survival and medical expenditure incurrence among subgroups (Figs. 1 and 2). In addition, the cost per life year was compared among subgroups. Although patients in the Surgery group paid higher costs of treatment in the initial stage of treatment, the costs of treatment per life year was the lowest, confirming that it was also important in terms of cost-effectiveness to diagnose lung cancer at low stage disease eligible for surgery. However, it should be taken to consideration that not all patients benefit from lung cancer screening and that the early detection and estimated cost-effectiveness of early detection and treatment could vary widely in the subgroups. The National Lung Screening Trial (NLST) study [12] showed that screening with low-dose CT was much more cost-effective in women than in men and in the groups with a higher risk of lung cancer than in those with a lower risk. A new report by Black et al., in 2019 [13] on the extended analysis of a patient cohort that was followed up after the initial NLST study stated that their original findings have been sustained. Therefore, the methods of detecting the high-risk group for lung cancer screening can affect the effectiveness of the screening and early detection and the high-risk group criteria are very important. In addition to the definition of the high-risk group based on the age and smoking history adopted by the NLST, it is necessary to develop a model for predicting lung cancer that reflects the risk factors for lung cancer, such as occupation, environmental factors, and family history.

In this study, we analysed lifetime survival and costs based on censored data using a semi-parametric extrapolation method developed by a research group in Taiwan [7]. The extrapolation method has already been validated by the research group [7, 14]. We also tested our semi-parametric extrapolated results with actual follow-up data in this study and confirmed that the results were validly estimated (Table A.1). Therefore, this study focused on assessing the burden of diseases rather than evaluating the validity of the research method.

The 5-year survival rate in this study was about 24% for overall patients, which in agreement with previous reports from the national statistics in South Korea. However, it is still the lowest survival rate compared to the average rate of other cancers [4]. Gong et al. reported that lung cancer showed the highest disability-adjusted life years (DALY) (594.6 DALYs/100,000 persons) among all cancers with DALY being higher for men (752.62 DALYs/100,000 persons) than for women (355.47 DALYs/100,000 persons) [15]. DALY is a representative indicator of disease burden, and it is a summed indicator of YLL due to premature death and years lost due to disability (YLD) from the disease. DALY was calculated using the metrics provided by the Global Burden of Disease Study group to estimate the disease burden of the entire population rather than at an individual level. DALY or YLD could be influenced more by subjective views and technical and theoretical weaknesses compared with YLL, which directly suggests the years lost due to the disease compared to a control group [16, 17]. YLL in this study were evaluated by comparing survival estimates between patients and controls using individual prescription data rather than values derived from the metrics based on national prevalence. Thus, YLL helps us more intuitively understand the loss of life caused by the lung cancer, and the burden of lung cancer could be evaluated more concretely when YLL at a patient level and DALY at the national level were presented together.

In addition, lead-time bias can occur if the earlier diagnosis had a significant effect on life extension [14, 18], and YLL can present more information on the outcome of a treatment modality without lead-time bias. For example, there was a difference in YLL and life expectancy between Surgery and CTx/RTx groups; the values were 16.5 years for Surgery group (YLL: 2.3, life expectancy: 14.2) and 15.2 years for CTx/RTx (YLL: 12.2, life expectancy: 3.0). The difference of 1.3 years between the groups can be described as a lead-time for the diagnosis of CTx/RTx group; however, this interpretation should be considered carefully as it is not a result of the same patient. The potential survival gain of 9.9 years calculated from YLL difference (YLL difference = 12.2–2.3 years) could be a survival indicator on adjusting for lead-time bias. This means that patients in the Surgery group survived approximately 10 years longer than patients in the CTx/RTx group, even after adjusting for lead-time bias. Thus, it can be inferred that it is advantageous to diagnose lung cancer as early as possible and perform a surgery to lower the disease burden.

To our knowledge, Yang et al.’s study is the only study that shows YLL results by comparing lifetime survival between patients and controls. The average life expectancy in their study was 3.05 years for non-small-cell lung cancer patients in the national cohort in Taiwan, and YLL were 11.84 for squamous cell carcinoma and 14.62 for adenocarcinoma. Direct comparisons are not possible because treatments and patient characteristics of the two studies were not identical; however, we assume that differences in patient selection and the method of extrapolation in both studies have affected the differences between the two studies significantly. In addition, the follow-up period before extrapolation could affect the results. The follow-up period of Yang’s study was 3–12 years depending on the patients’ enrolment period, whereas that of this study was fixed to 100 months (about 8.3 years) for all patients. According to the sensitivity analysis of this study, if the follow-up period were to be shortened from 100 months to 60 months, the lifetime expectancy would be reduced by 0.9 years in the total patients and by 3.0 years in the Surgery group.

Generally, the aim of a disease burden study is to quantify premature mortality and disability. Furthermore, it would help prioritise healthcare resources depending on economic appraisal of the disease burden [19]. Therefore, this study included YMC results to compare outcomes of cost per survival. The YMC of Surgery+CTx/RTx and CTx/RTx groups was 1.62 times and 1.75 times higher than that of the Surgery group, respectively. Comparing cost ratio according to treatment patterns could be a more useful reference for other countries because absolute treatment costs vary greatly country due to different social settings for medical environments, surgery costs and other factors. A study using US health claim data reported similar results as this study. Patients in stage I with non-small-cell lung cancer showed the lowest medical cost per survival month, and the costs for patients in stages II, IIIA, IIIB and IV increased by about 1.41, 1.55, 2.41 and 2.96 times of stage I, respectively [20].

The dramatic improvements in lung cancer outcomes can be attributed to the recent advances in novel targeted therapy and immunotherapy [21]. As the treatment paradigm for lung cancer evolves, concerns about the rising cost of these novel therapeutics have become a global dilemma. Considering that immunotherapy and most targeted therapy agents were not allowed in the National Health Insurance Service during this study period, we can predict that the cost of treatment of patients with advanced disease will increase dramatically after the next decade. Furthermore, targeted and immunotherapy agent related toxicities are more tolerable compared to the platinum-based chemotherapy agents. The proportion of the chemotherapy group is expected to gradually increase in the elderly patient population, which might be another social cost of the disease. With the rapid increase in the elderly population and complex chronic diseases, the treatment strategy is shifting to selecting a treatment plan that can bring about better outcome for the patient while paying the same cost, and evaluation of the economic efficiency of each treatment method become more and more important [22, 23]. Though this study, we expect that life expectancy, YLL and cost data could be derived using a semi-parametric method based on a real claim database, and they are likely to contribute to assessing the cost-effectiveness of new treatment options with less uncertainty [13].

This study has the following limitations. First, the prognosis and surgical decisions of cases of lung cancer are closely related to the histology, stage, patient performance and molecular subtype; however, our database did not include such information, and by not adjusting for such confounding factors, our analysis may include biased outcomes. In other studies involving analyses using claim data, treatment pattern was provided to give further information on the disease stage by categorising the patients with treatment regimen or baseline metastasis [24, 25]. Therefore, this study also conducted subgroup analysis according to the treatment modality to provide additional information on the overall results of patients. However, the results should be interpreted carefully considering that a treatment modality does not suggest a specific stage and palliative radiation and curative stereotactic radiation could not be clearly differentiated. Second, due to the limitation of source data, this study could not provide results based on histological types, such as non-small cell lung cancer and small cell lung cancer, which may have different YLL and medical expenditure. Since about 85% of lung cancer patients in South Korea have non-small cell type cancer [26]. the results of this study may be more biased toward non-small cell lung cancer. Third, there may have been changes in treatment patterns and treatment unit cost in the 2004–2010 period; however, these changes were not considered in our analysis. Instead, overall price level was adjusted in line with the expenses of 2017 to minimise the impact of price changes. Forth, social costs, such as out-of-pocket money and loss of productivities, were not included. In many other studies on the costs of lung cancer treatment, the out-of-pocket expenses were not considered due to their high uncertainty [27]. As of 2015, the coverage of health insurance by Korean government for cancer patients was 76% [28]; therefore, the total medical expenses actually paid by patients would be 1.3 times higher than reported in this study.

Nevertheless, this study has strong points that for the first time, various indicators of lung cancer burden were presented at an individual level over lifetime horizon in South Korea based on real-patient data. The results would be useful in planning cost-effective prevention and treatment policies for lung cancer and could also be a reference for other countries in similar environments as Korea.

## Conclusions

Lung cancer has resulted in YLL of about 10 years compared with the general population, and the treatment modality has an association with the disease burden indicators. Early diagnosis in patients with low stage disease eligible for surgery and timely treatment seem to be very important and cost effective strategies for lung cancer treatment considering the lowest YLL and YMC in the Surgery group.

## Supplementary information

**Additional file 1: Figure A.1.** Target subject selection scheme. **Table A.1.** Comparison of 10-year survival estimates between the semi-parametric extrapolation method (5-year follow-up and 5-year extrapolation) and the Kaplan–Meier method (10-year follow-up). **Table A.2.** Sensitivity analysis in the lifetime of 20 years. **Table A.3.** Sensitivity analysis in the lifetime of 30 years.

## Data Availability

The datasets generated and/or analysed during the current study are not publicly available because the Korean National Health Insurance Sharing Service (KNHISS) does not allow researchers to provide data personally or share publicly but are available from the corresponding author on reasonable request.

## Abbreviations

BSC: Best supportive care
CTx/RTx: Chemo and/or radiotherapy
DALY: Disability-adjusted life years
RTx: Radio-therapy
SE: Standard error
YLL: Years of life lost
YLD: Years lost due to disability
YMC: Yearly medical cost
