# Supplementary material for: Lifetime survival and medical costs of lung cancer: a semi-parametric estimation from South Korea
Source: BMC Cancer. 2020 Sep 3;20:846. doi: 10.1186/s12885-020-07353-8 (PMC7650522; doi:10.1186/s12885-020-07353-8)
Supplement: Supplementary file 1 — Additional file 1: Figure A.1. Target subject selection scheme. Table A.1. Comparison of 10-year survival estimates between the semi-parametric extrapolation method (5-year follow-up and 5-year extrapolation) and the Kaplan–Meier method (10-year follow-up). Table A.2. Sensitivity analysis in the lifetime of 20 years. Table A.3. Sensitivity analysis in the lifetime of 30 years. [file 12885_2020_7353_MOESM1_ESM.docx]

**Supplementary Appendix**

Supplement to: Park HY et al., Lifetime survival and medical costs of lung cancer: A semi-parametric estimation from South Korea.

**Figure A.1. Target subject selection scheme**


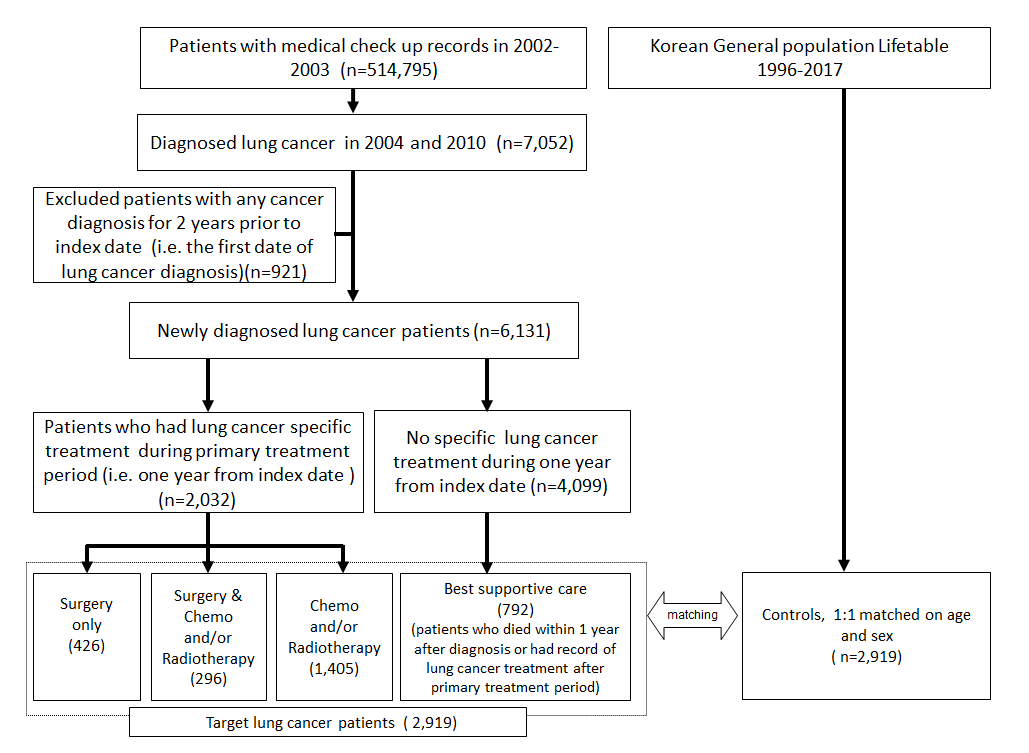


**Table A.1. Comparison of 10-year survival estimates between the semi-parametric extrapolation method (5-year follow-up and 5-year extrapolation) and the Kaplan–Meier method (10-year follow-up).**

|  | Number of patients | | | 10-year survival estimates (year) | | | | Ratio  A to B  (%) |
| --- | --- | --- | --- | --- | --- | --- | --- | --- |
|  | Month 0 | Month 60 | Month 120 | (A) Semi-parametric extrapolation method (5-year follow-up + 5-year extrapolation) | | (B) Kaplan–Meier method (10-year follow-up) | |  |
|  |  |  |  | Mean | (SE) | Mean | (SE) |  |
| Total | 2,919 | 634 | 115 | 2.69 | 0.08 | 2.76 | 0.07 | 97.4% |
| Surgery | 426 | 329 | 69 | 7.65 | 0.25 | 7.82 | 0.17 | 97.8% |
| Surgery+CTx/RTx | 296 | 153 | 18 | 5.88 | 0.25 | 5.73 | 0.23 | 102.7% |
| CTx/RTx | 1,405 | 152 | 28 | 1.87 | 0.06 | 2.00 | 0.07 | 93.5% |

CTx/RTx; chemotherapy and/or radiation therapy

**Table A.2. Sensitivity analysis in the lifetime of 20 years**

| Survival: year (SE), Cost: USD (SE) | Follow-up period | Survival time (ST) | | Medical expenditure (ME) | | Cost per life year in USD |
| --- | --- | --- | --- | --- | --- | --- |
|  |  | Actual ST during real follow-up | Estimated ST for lifetime (20 years) | Actual ME during real follow-up | Estimated ME for lifetime (20 years) |  |
| Total (n = 2,919) | 100 (base) | 2.48 (0.06) | 4.11 (0.15) | 22,133 (489) | 28,962 (971) | 5,315 (338) |
|  | 60 | 1.82 (0.04) | 3.52 (0.17) | 19,546 (406) | 28,813 (1,051) | 6,820 (491) |
|  | 80 | 2.16 (0.05) | 4.12 (0.17) | 20,925 (381) | 29,432 (893) | 5,743 (356) |
|  | 120 | 2.76 (0.07) | 4.09 (0.18) | 22,886 (542) | 27,354 (948) | 4,773 (364) |
|  | Last (143 month) | 3.07 (0.08) | 4.26 (0.17) | 23,610 (608) | 27,656 (1,172) | 4,646 (371) |
| Surgery (n = 426) | 100 (base) | 6.73 (0.13) | 12.77 (0.62) | 30,846 (1,200) | 55,141 (3,769) | 4,328 (361) |
|  | 60 | 4.32 (0.07) | 10.86 (1.25) | 22,554 (870) | 51,435 (4,023) | 4,816 (661) |
|  | 80 | 5.56 (0.09) | 12.57 (0.63) | 27,265 (1,127) | 57,097 (4,055) | 4,561 (394) |
|  | 120 | 7.82 (0.17) | 12.81 (0.60) | 33,275 (1,324) | 49,491 (3,524) | 3,870 (328) |
|  | Last (143 month) | 9.03 (0.21) | 13.26 (0.50) | 36,888 (1,697) | 49,491 (3,524) | 4,187 (424) |
| Surgery+CTx/RTx (n = 296) | 100 (base) | 5.09 (0.20) | 8.02 (0.80) | 42,385 (1,946) | 57,747 (3,950) | 7,110 (864) |
|  | 60 | 3.56 (0.10) | 9.04 (0.90) | 35,830 (1,465) | 66,026 (4,796) | 7,338 (898) |
|  | 80 | 4.39 (0.13) | 8.47 (0.79) | 39,409 (1,549) | 61,931 (4,573) | 7,279 (868) |
|  | 120 | 5.73 (0.23) | 8.59 (0.76) | 45,035 (2,451) | 56,033 (5,129) | 6,402 (827) |
|  | Last (137 month) | 6.20 (0.29) | 8.86 (0.61) | 45,970 (2,433) | 53,878 (4,451) | 5,944 (650) |
| CTx/RTx (n = 1,405) | 100 (base) | 1.87 (0.06) | 2.75 (0.15) | 22,255 (654) | 28,914 (1,076) | 7,664 (651) |
|  | 60 | 1.56 (0.05) | 2.00 (0.10) | 24,243 (499) | 27,679 (805) | 10,135 (744) |
|  | 80 | 1.73 (0.06) | 2.61 (0.21) | 24,684 (603) | 28,195 (1,100) | 8,124 (925) |
|  | 120 | 2.00 (0.07) | 2.59 (0.15) | 25,443 (633) | 27,509 (886) | 7,159 (668) |
|  | Last (142 month) | 2.13 (0.08) | 2.67 (0.16) | 25,513 (661) | 26,262 (779) | 6,532 (636) |

CTx/RTx; chemotherapy and/or radiation therapy

^a^ Applied exchange rate: 1,100 Korea Won/USD

**Table A.3. Sensitivity analysis in the lifetime of 30 years**

| Survival: year (SE), Cost: USD (SE) | Follow-up period | Survival time (ST) | | Medical expenditure (ME) | | Cost per life year in USD |
| --- | --- | --- | --- | --- | --- | --- |
|  |  | Actual ST during real follow-up | Estimated ST for lifetime (30 years) | Actual ME during real follow-up | Estimated ME for lifetime (30 years) |  |
| Total (n = 2,919) | 100 (base) | 2.48 (0.06) | 4.79 (0.26) | 22,131 (417) | 32,139 (1,246) | 5,442 (445) |
|  | 60 | 1.82 (0.04) | 3.77 (0.23) | 19,545 (413) | 30,251 (1,241) | 6,849 (576) |
|  | 80 | 2.16 (0.05) | 4.75 (0.26) | 20,926 (427) | 32,499 (1,274) | 5,796 (453) |
|  | 120 | 2.76 (0.07) | 4.79 (0.28) | 22,886 (424) | 30,011 (1,165) | 4,881 (433) |
|  | Last (143 month) | 3.07 (0.08) | 5.29 (0.30) | 23,612 (589) | 32,033 (1,880) | 4,875 (491) |
| Surgery (n = 426) | 100 (base) | 6.73 (0.13) | 15.14 (1.22) | 30,836 (1,237) | 66,093 (5,477) | 4,393 (503) |
|  | 60 | 4.32 (0.07) | 11.43 (2.01) | 22,553 (981) | 54,652 (7,281) | 4,942 (1,055) |
|  | 80 | 5.56 (0.09) | 14.70 (1.56) | 27,270 (1,007) | 67,514 (6,825) | 4,650 (674) |
|  | 120 | 7.82 (0.17) | 15.27 (1.11) | 33,269 (1,402) | 58,821 (6,430) | 3,872 (506) |
|  | Last (143 month) | 9.03 (0.21) | 16.08 (0.87) | 36,888 (1,847) | 70,576 (7,487) | 4,393 (522) |
| Surgery+CTx/RTx (n = 296) | 100 (base) | 5.09 (0.20) | 8.82 (1.20) | 42,377 (2,265) | 62,380 (6,299) | 7,066 (1,194) |
|  | 60 | 3.56 (0.10) | 10.47 (1.50) | 35,830 (1,532) | 74,674 (7,894) | 7,253 (1,272) |
|  | 80 | 4.39 (0.13) | 9.49 (1.27) | 39,408 (1,590) | 68,162 (6,765) | 7,236 (1,196) |
|  | 120 | 5.73 (0.23) | 10.09 (1.26) | 45,035 (2,517) | 62,254 (7,095) | 6,141 (1,041) |
|  | Last (137 month) | 6.20 (0.29) | 11.02 (1.17) | 45,970 (2,297) | 60,014 (5,118) | 5,402 (740) |
| CTx/RTx (n = 1,405) | 100 (base) | 1.87 (0.06) | 3.20 (0.22) | 25,255 (574) | 30,931 (1,293) | 7,581 (755) |
|  | 60 | 1.56 (0.05) | 2.00 (0.12) | 24,246 (502) | 27,739 (931) | 10,166 (937) |
|  | 80 | 1.73 (0.06) | 2.90 (0.30) | 24,684 (621) | 29,453 (1,422) | 8,042 (1,152) |
|  | 120 | 2.00 (0.07) | 2.88 (0.28) | 25,444 (652) | 28,681 (1,221) | 7,215 (1,022) |
|  | Last (142 month) | 2.13 (0.08) | 3.17 (0.25) | 25,513 (579) | 26,932 (746) | 6,176 (679) |

CTx/RTx; chemotherapy and/or radiation therapy

^a^ Applied exchange rate: 1,100 Korea Won/USD
